# Spatial-temporal clustering of notified pulmonary tuberculosis and its predictors in East Gojjam Zone, Northwest Ethiopia

**DOI:** 10.1371/journal.pone.0245378

**Published:** 2021-01-15

**Authors:** Mulusew Andualem Asemahagn, Getu Degu Alene, Solomon Abebe Yimer

**Affiliations:** 1 School of Public Health, College of Medicine and Health Sciences, Bahir Dar University, Bahir Dar, Ethiopia; 2 Faculty of Medicine, Department of Microbiology, Unit for Genome Dynamics, University of Oslo, Oslo, Norway; 3 Coalition for Epidemic Preparedness Innovations (CEPI), Oslo, Norway; University of South Carolina, UNITED STATES

## Abstract

**Background:**

Tuberculosis (TB) remains a key health menace in Ethiopia and its districts. This study aimed to assess the spatial-temporal clustering of notified pulmonary TB (PTB) cases in East Gojjam Zone, Northwest Ethiopia.

**Methods:**

A retrospective study was conducted among all PTB cases reported from 2013–2019. Case notification rates (CNRs) of PTB cases at *Kebele* (the lowest administrative unit), *woreda*, and zone levels were estimated. The PTB clustering was done using global Moran’s I statistics on Arc GIS 10.6. We used Kulldorff SaTScan 9.6 with a discrete Poisson model to identify statistically significant spatial-temporal clustering of PTB cases at *Kebele* level. Similarly, a negative binomial regression analysis was used to identify factors associated with the incidence of PTB cases at kebele level.

**Results:**

A total of 5340 (52%) smear-positive and 4928 (48%) smear-negative PTB cases were analyzed. The overall mean CNR of PTB cases at zone, *woreda* and *Kebele* levels were 58(47–69), 82(56–204), and 69(36–347) per 100,000 population, respectively. The purely spatial cluster analysis identified eight most likely clusters (one for overall and one per year for seven reporting years) and 47 secondary clusters. Similarly, the space-time scan analysis identified one most likely and seven secondary clusters. The purely temporal analysis also detected one most likely cluster from 2013–2015. Rural residence, distance from the nearest health facility, and poor TB service readiness were factors (p-value <0.05) to PTB incidence at *kebele* level.

**Conclusion:**

The distribution of PTB cases was clustered. The PTB CNR was low and showed a decreasing trend during the reporting periods. Rural residence, distance from the health facilities, and poor facility readiness were factors of PTB incidence. Improving accessibility and readiness of health facilities mainly to rural and hotspot areas is vital to increase case detection and reduce TB transmission.

## Introduction

Tuberculosis is a leading killer of infectious diseases globally. The burden of TB highly varies among countries from fewer than five to over 500 new TB cases per 100,000 population per year [1]. Over three million globally estimated new TB cases are not yet reported due to underreporting of detected cases and/or underdiagnosis. The majority of 20 high TB, TB-HIV and multi-drug resistant (MDR-TB) burden countries are from Africa that accounted for a quarter of the world’s TB caseloads [1].

Despite continuous TB prevention and control efforts, Ethiopia is still among the 14 high TB, TB-HIV and MDR-TB burden countries. There were 156 800 estimated new TB cases, 24 080 deaths, 1456 MDR-TB cases and 10 192 TB-HIV coinfected cases in 2019 [1]. Research findings from different parts of Ethiopia [2–6] showed the presence of high TB burdens with varied magnitude across its regions and *woredas* (third level administrative hierarchy in Ethiopia). Recent studies that used the applications of geographic information system (GIS) in the analysis of TB data exhibited that TB incidence and prevalence in Ethiopia have spatial and space-time variations [7–11].

The presence of inadequate evidence on the spatial-temporal distribution of disease might be a contributing factor for the lack of knowledge on identifying areas with high TB incidence (hotspots) and driving factors for TB clustering. Understanding the spatiotemporal distribution of TB helps policymakers and TB control program managers to design proper plans for TB control and efficiently undertaking targeted TB control interventions [8,10,11].

Amhara Region, the second largest and populous region in Ethiopia, has contributed more to the national TB burden [9,12–14]. The average TB CNR of all forms of TB in the region was 107 per 100 000 population ranging from 18 to 614 per 100 000 population [9]. Studies conducted in different parts of the region showed various proportions of TB burden [2,14–17].

There were a few previous studies on the spatial-temporal distribution of TB in some parts of the Amhara region [9,11,18]. They were based on the national and regional TB reports which missed important variables of TB cases (actual living places/*kebele*s of TB cases) compared to using TB records from unit TB registration books of health facilities. There might also be miss matching between facility registers and national reports [19]. In addition, the previous studies tried to identify high TB burden areas (hotspots) and associated factors at region, zone and *woreda* levels using the 2007 population census and variables, which is too old to represent the current situation. This situation has affected the performance of TB prevention and control programs and the allocation and efficient utilization of limited resources available for TB.

This study aimed to assess the spatial, temporal, and space-time cluster of notified PTB cases at *Kebele* levels (actual living places of PTB cases) in East Gojjam Zone of the Amhara Region, northwest Ethiopia. We selected the study area because of: 1) The diverse geographic features that the zone has which might limit access to TB diagnosis and treatment centers. Thus, PTB cases might be clustered in certain geographic locations and applying uniform TB control measures might not be effective in extenuating the problem. 2) The high TB burdens and the least TB CNR (46%) from all zones in the region [12,20]. 3) Many people have poor access to TB diagnostic and treatment services mainly due to a lack of laboratory staff, TB diagnostic equipment, reagents, and supplies [21,22]. 4) Based on our search, there is no former study on the spatial-temporal distribution of TB cases in East Gojjam Zone.

## Materials and methods

### Study design and settings

**Fig 1:**
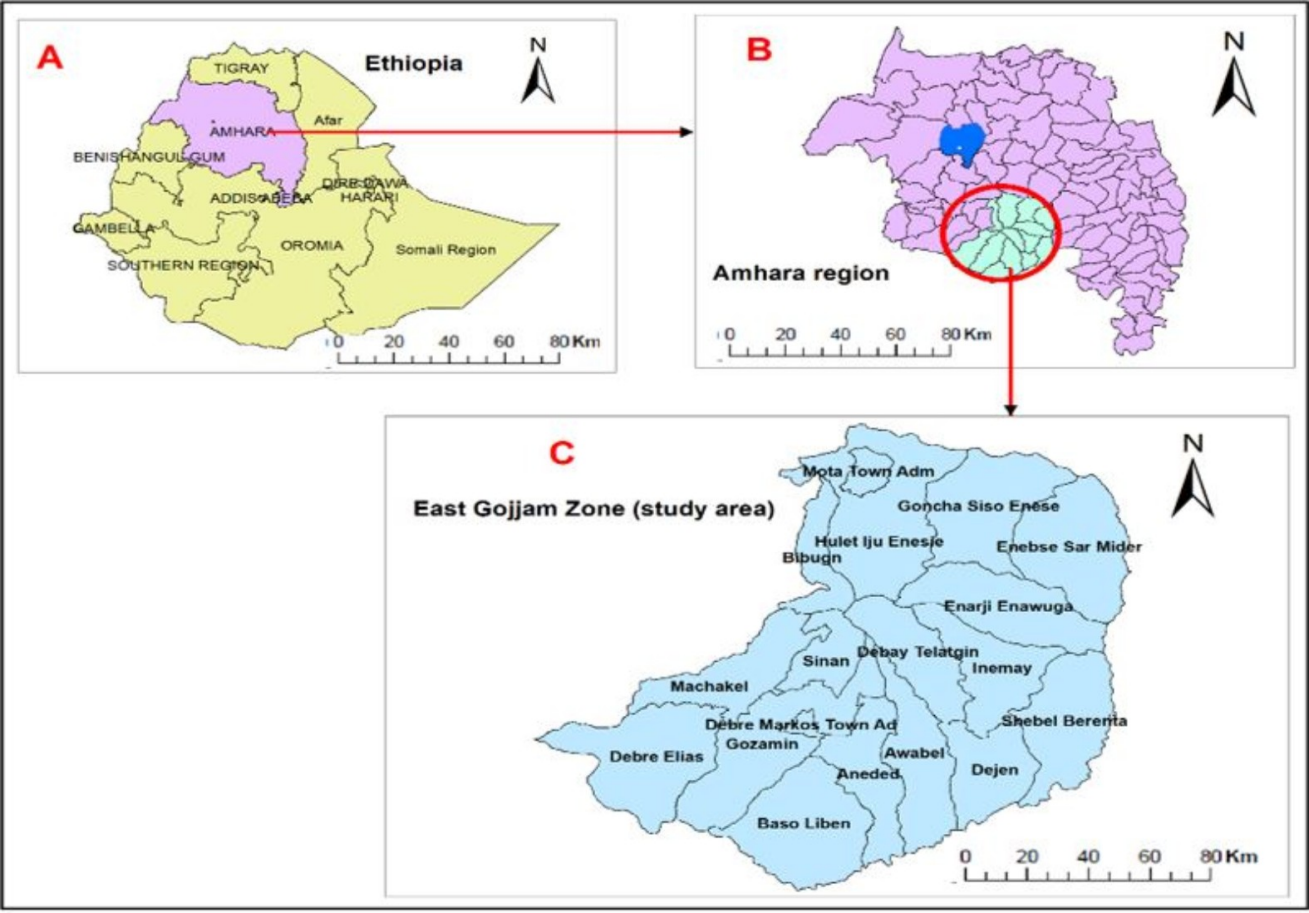
Map of East Gojjam Zone, Northwest Ethiopia, 2019 (Coordinates: 9.900° to 11.193° latitude, and 37.152° to 38.489° longitude).

A retrospective spatial-temporal study was conducted from June to December 2019 using records of notified PTB cases during 2013–2019 in the East Gojjam Zone, one of the 12 zones of the Amhara Region, Ethiopia. East Gojjam Zone is located between 9.900° to 11.193° latitude, and 37.152° to 38.489° longitude [23]. The Zone is about 300 km to the northwest of Addis Ababa and 265 km from Bahir Dar, a capital city of Amhara region. There was an estimated population of 2,632,632 (2,237,737 rural and 394, 895 urban) in 2018 [12]. Over 85% of the study area population is living in rural settings. It has about 14,010 km^**2**^ area coverage divided into 18 administrative *woredas* which are further divided into 49 urban and 392 rural *Kebeles*. East Gojjam Zone is bounded by the Nile river and has diverse geographic features: *kola* (lowlands), *dega* (mountainous), *woina-dega* (plateau), and the Nile river valleys that might affect access and quality of TB control activities [12,15]. The zone’s climate has four seasons: summer (a rainy season in Ethiopia from June to August), autumn (September to November), Bega/winter (a dry season in Ethiopia lasting from December to February), and spring (March to May) [9]. Based on the Amhara Regional health bureau report [12], there were 120 (102 health centers, nine hospitals, and nine private clinics) offering TB services in 2018 (**Fig 1**).

### Data collection sources and procedures

We included records of all PTB patients who attended their anti-TB treatments during 2013–2019. Ten trained data collectors (nurses, health officers, and health information technicians) extracted the PTB records from the unit TB registers using a pretested data extraction tool. The tool is comprised of variables including age, sex, patient treatment category, address, residence, date of treatment start, treatment outcome, type of diagnosis, forms of TB, family TB history and comorbidity status (HIV/AIDS and diabetes mellites). Data from health facilities that have an electronic recording system were exported to the excel format. Whereas, a manual data extraction process was employed to collect data from health facilities that have only manual TB registration logbooks. About 147 (1.4%) of PTB patient records were excluded during data collection since they were residing outside of the study Zone. In addition, data on the population and geographic coordinates of each *Kebele* were obtained from the Amhara Regional state plan commission Office that was based on the central statistical agency projection [23]. We also computed the population density of each *Kebele* by dividing the population of each *Kebele* with the total area coverage of each *Kebele*. Moreover, the readiness of all health facilities, the ability of health facilities to offer TB diagnostic and treatment services, was assessed using the WHO and Ethiopian service availability and readiness assessment tools [24,25]. The tool used to measure facility readiness has16 tracer items focusing on availability of trained staff, diagnostic and treatment guidelines, diagnostics, equipment, laboratory supplies, and anti-TB drugs. Distance from each *kebele* to the nearest health facility (elucidation distance) on the other hand was measured using GIS 10.6. The detailed procedures of measuring facility readiness and their distance from each *kebele* were stated by our former paper conducted in the study area [21].

### Data quality assurance

We gave three days of training to data collectors and supervisors. The data extraction guideline was pretested before actual data collection was started. Data collectors also used a data collection manual. PTB records not having addresses information and cases from outside of the study area were excluded. In addition to checking data completeness and consistency, supervisors and investigators cross-checked data for each PTB case with the quarterly and annual TB reports of the facility, *woreda*, zone and Amhara Regional State Health Bureau. Data were carefully entered into the 2019 Microsoft excel format and exported to SPSS version 25 for further cleaning and analysis. Double data entry was done into Microsoft Excel format. Each TB record together with its Kebele was carefully geocoded based on address information from unit TB registers and using X and Y-coordinates (S1 File). All *Kebele* attributes (residence, PTB cases, population, population density, distance from the nearest health facility, CNR and coordinates) were properly recorded and crosschecked before analysis. We checked all the necessary assumptions for our data analysis.

### Data processing and analysis

The overall TB service readiness score was computed by taking the mean value of the core domain (trained staff and diagnostic and treatment guidelines, diagnostics, anti-TB drugs, and laboratory supplies) scores derived from a total of 16 tracer items. Thus, health facilities that scored below the mean were categorized as poor readiness (coded by 1) and others that scored the mean and above were stated as good readiness (coded by 2) in the analysis [7]. Similarly, a 10km distance, a reference standard used by the Ethiopian ministry of health to measure access to health facilities, was used as a cut-off value to measure the distance of each *kebele* to the nearest health facility. Accordingly, areas within 10km distance from the nearest health facilities were categorized as had good access to health facilities (coded by 1), and those areas located beyond a 10km radius were categorized as had poor access to health facilities (coded by 2) [21,26]. Moreover, the population density of each *kebele* was grouped into two categories: areas with a population density less than the national population density/115/km2/(coded by 1) and areas greater than the national average population density (coded by 2).

The extracted PTB records were entered into Microsoft excel. The mean and annualized PTB case notification rates (CNRs) were estimated at zone, *woreda* and *Kebele* levels by dividing the total number of PTB cases over the population of a given year for the zone, *woreda*, and *Kebele* and multiplied by 100,000 population at risk. We also prepared an attribute table for *Kebeles* as mentioned under the quality assurance section. All the PTB records were geocoded based on their X and Y-coordinates of each *Kebele* where PTB cases were living using the ArcGIS 10.6 (S1 File). Spatial empirical Bayes smoothing was applied in geographic data analysis tools (GeoDa) to overcome a variance instability in small areas, which is due to differences in population size, as well as few PTB cases in some areas [8]. About 253 (2.3%) of PTB cases were excluded from the analyses due to missing data on address information.

### Negative binomial regression analysis

After checking the assumptions for Poisson regression analysis, we found that PTB data were over dispersed since the ratio of deviance to the degree of freedom was greater than 1, and the mean value of PTB data was less than the variance value. Consequently, we chose a negative binomial regression analysis under generalized linear models to identify factors associated with the kebele level incidence rate of PTB. Accordingly, we did a negative binomial regression analysis in three ways to choose the best model with the lowest Akaike’s Information Criteria (AIC) and Bayesian Information Criteria (BIC) value. First, we did a negative binomial regression analysis by choosing "negative binomial with log link" from the type of model window. Second, a negative binomial regression analysis by clicking on a "custom tab" from the type of model window. In this step, we used a negative binomial model from options under "Distribution", Log from link function, and click on the "specify value 1 under the box". Third, we followed the procedures sated under the secondary step, except click on the "Estimate values" instead of "specify value 1". The covariates were statistically significant in all steps with similar signs under the coefficient (B) except having different values for AIC and BIC. Finally, we chose the last model (third) since it showed the least AIC and BIC values. The statistical significance was determined by p-value < 0.05 and the association was described using Exp(B) at 95%CI.

### Spatial autocorrelation analysis

The global Moran's I statistic was used in ArcGIS 10.6 to examine the presence of spatial clustering of PTB cases in the study area. The significance of Moran’s I is identified based on the Monte Carlo method, which simulates the distribution according to random data derived from Pearson’s correlation coefficients. The Moran’s I value greater than zero and p-value < 0.05 shows clustering. Whereas, Moran’s I value zero shows no spatial autocorrelation (the pattern is random) and less than zero shows the pattern is dispersed. A spatial weight matrix was used to specify the spatial relationships of the *Kebeles*. Neighbors were defined using the first-order queen’s polygon contiguity and *Kebeles* sharing borders/common vertex were considered neighbors. The local locations of PTB clusters at *kebele* levels were indicated through Kulldroff’s scan statistic using a SaTScan 9.6 [7,8,10].

### Purely spatial, temporal and space-time analysis

A Kulldroff’s scan statistic using a SaTScan 9.6 was used to detect the size and locations of purely spatial, temporal and space-time clusters using the number of PTB cases, population size, year of TB diagnosis, and the geo-coordinates data at *Kebele* levels. Kulldroff’s scan statistics is a widely used tool for spatial, temporal, and space-time cluster analysis for diseases in different settings. It also computes the likelihood ratio (LLR), relative risk (RR) and provides a p-value using Monte Carlo Simulations [27]. Scan circles of various sizes were used to identify the most likely purely spatial, temporal, and space-time clusters of PTB cases. The scanning window was a time interval in the purely temporal, a circle in the purely spatial, and a cylinder in spatial-temporal with a circular geographic base representing space and height for the time-period. The upper limit for the maximum purely spatial and space-time cluster size was set to be 50% of the population at risk, which is important to detect small and large clusters. Similarly, the maximum time was specified to be 50% within the study period for purely temporal cluster analysis [27]. In the space-time cluster analysis, it assumes that the RR of PTB was the same within the window compared to the outside [8]. The time of the space-time cluster analysis was set to the time frame from 2013–2019.

The purely spatial, temporal, and space-time clusters were selected based on the likelihood ratio and p-value < 0.05. The LLR was calculated to measure a RR of PTB occurrence within the cluster when compared to the risk outside using Monte Carlo Simulations. The maximum number of permutations for Monte Carlo simulation was set to 9999. The cluster with the maximum LLR was defined as the most likely cluster (a cluster with the highest LLR test) means a cluster least likely to have occurred by chance). Secondary clusters are clusters that are in the rank order after primary clusters by their LLR test statistic. A standard of no geographical overlap, and hierarchical cluster type were selected to report secondary clusters (separation of clusters). Statistical significance for the presence of clusters was reported when a P-value was < 0.05 [28].

## Ethical considerations

This study was conducted following the declaration of Helsinki. The ethical review committee of the College of Medicine and Health Sciences, Bahir Dar University reviewed the research protocol and gave an ethical clearance (protocol No: 091/18-04). The Amhara Regional State Health Bureau and East Gojjam Zone Health Department gave supporting letters and consent to collect data since it was a secondary data set owned by them. Patient records were de-identified/made anonymous using codes to keep data confidentiality.

## Results

### Demographic characteristics of PTB cases

A total of 19,446 TB cases, 10,668 PTB (5,547 bacteriologically confirmed and 5,121 smear-negative) PTB cases, and 8,778 extrapulmonary TB were diagnosed during the period from 2013 to 2019 in the study area. We considered only PTB cases in our analysis since untreated PTB cases are the primary sources of TB infection. We excluded 400(3.7%) PTB cases from the analysis due to no address information and their permanent residences are outside of the Zone. Thus, a total of 10,268 PTB cases, 5,751(56%) male and 4,517(44%) females, were used for spatial-temporal analysis. Over half, (52%) of PTB cases were smear-positive. The mean age with a standard deviation of cases was 34±6 years. Over half, (54%) of PTB cases were from rural areas. Of 7,140 PTB cases who have known HIV status, 925 (9%) were HIV positive. The treatment success rate ranged from 87% in 2013 to 94% in 2019 (**Table 1**).

**Table 1:** Characteristics of PTB cases from 2013–2019 in East Gojjam Zone, Ethiopia.

Variable	2013 N (%)	2014 N (%)	2015 N (%)	2016 N (%)	2017 N (%)	2018 N (%)	2019 N (%)	2013–2019 N (%)
Age in years								
≤ 14	112 (7)	155 (9)	110 (7)	120 (8)	110 (8)	127 (10)	124 (10)	858(8.3)
15–34	776 (48)	687 (40)	704 (45)	671 (45)	633 (46)	633 (50)	608 (49)	4712(46.0)
35–44	453 (28)	584 (34)	423 (27)	447 (30)	248 (18)	304 (24)	236 (19)	2695(26.2)
45+	274 (17)	290 (17)	327 (21)	252 (17)	385 (28)	203 (16)	272 (22)	2003(19.5)
Sex								
Male	905 (56)	961 (56)	860 (55)	835 (56)	785 (57)	696 (55)	707 (57)	5751(56.0)
Female	711 (44)	755 (44)	704 (45)	655 (44)	591 (43)	569 (45)	533 (43)	4517(44.0)
Residence								
Rural	873 (54)	944 (55)	829 (53)	805 (54)	730 (53)	684 (54)	682 (55)	5545(54.0)
Urban	743 (46)	772 (45)	735 (47)	685 (46)	646 (47)	582 (46)	558 (45)	4723(46.0)
Type of TB								
Smear positive PTB	857 (53)	893 (52)	814 (52)	760 (51)	716 (52)	659 (52)	641(52)	5340(52.0)
Smear negative PTB	759 (47)	823 (48)	750 (48)	730 (49)	660 (48)	607 (48)	599 (48)	4928(48.0)
TB category								
New	1583 (98)	1664 (97)	1517 (97)	1460 (98)	1334 (97)	1236 (98)	1215 (98)	10008(97.5)
Retreatment	36 (2.2)	52 (3)	47 (3)	30 (2)	42 (3.1)	28 (2.2)	25 (2)	260(2.5)
HIV status								
Positive	178 (11)	172 (10)	141 (9)	134 (9)	124 (9)	102 (8)	87 (7)	938(9.0)
Negative	1438 (89)	1544 (90)	1423 (91)	1356 (91)	1252 (92)	1164 (92)	1153 (93)	9330(91.0)
Has TB contact history								
Yes	485 (30)	549 (32)	438 (28)	358 (24)	248 (18)	253 (20)	273 (16)	2604(25.4)
No	1131 (70)	1167 (68)	1126 (72)	1132 (76)	1183 (86)	1013 (80)	967 (78)	7664(74.6)
Treatment outcome								
Cured	760 (47)	686 (40)	657 (42)	656 (44)	601 (42)	532 (42)	546 (44)	4438(43.2)
Completed	646 (40)	841 (49)	750 (48)	715 (48)	716 (50)	646 (51)	620 (50)	4934(48.0)
Defaulted	73 (4.5)	69 (4.0)	63 (4.0)	37 (2.5)	43 (3.0)	38 (3.0)	25 (2.0)	348(3.4)
Failure	75 (6.0)	73 (5.0)	63 (4.0)	56 (4.0)	48 (4.0)	31 (2.5)	30 (3.0)	376(3.7)
Relapse	40 (2.5)	34 (2.0)	31 (2.0)	22 (1.5)	14 (1.0)	19 (1.5)	12 (1.0)	172(1.7)
** Total**	1594	1703	1564	1486	1422	1266	1233	10,268

### Spatial distribution of PTB at *woreda* and *Kebele* levels

**Fig 2:**
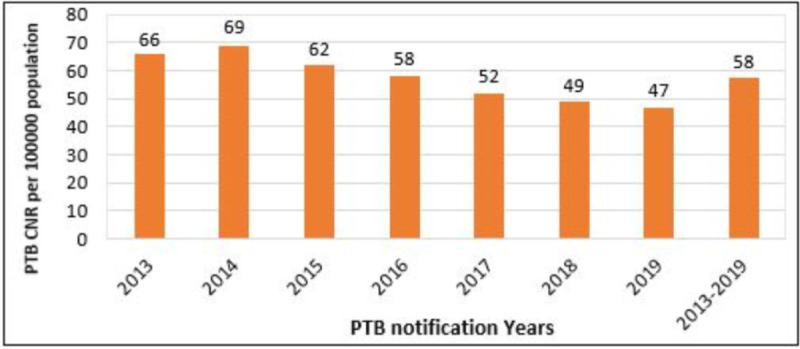
PTB Case notification rate per 100,000 population in East Gojjam Zone, 2013–2019.

The annual PTB CNR varied by *woredas* and *Kebeles* over the reporting years. The overall average annual PTB CNR from 2013–2019 was 58 (range: 47–69 per 100,000) people. (**Fig 2**).

**Fig 3:**
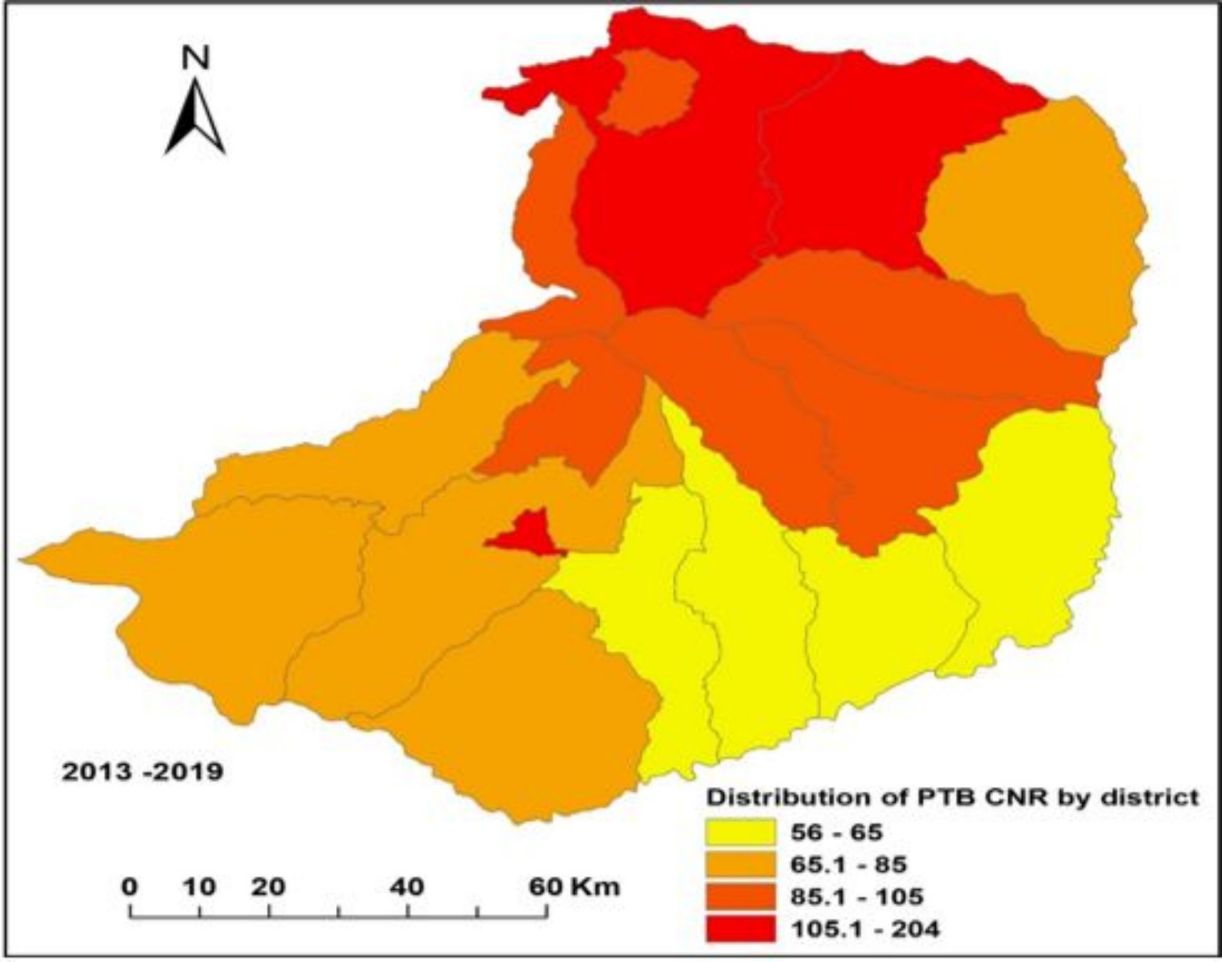
Distribution of PTB CNR per 100000 population in East Gojjam Zone, 2013–2019 (Coordinates: 9.900° to 11.193° latitude, and 37.152° to 38.489° longitude).

The annual CNR at the *woreda* level was 82 (range: 56–204 PTB cases) per 100,000 population where the highest CNR was from *kebele*s of Debremarkos town administration and the lowest was from *kebele*s of Shebel Berenta and Dejen *woredas*. Most of the highest PTB cases during the reporting periods were from the northern and central parts of the study area (**Fig 3**).

The average annual CNR was also varied by *Kebele* and gender. The PTB CNR at *Kebele* levels was 69 (range: 36–347) per 100,000 population. It was also higher among males (31/100 000 population) than females (26/100 000 population) during the reporting periods (**Table 1)**.

### Spatial autocorrelation analysis

The global Moran's I statistic was significant for each year, indicating that the distribution pattern of PTB cases was clustered in the study area. The annual Moran’s I values were positively autocorrelated and indicated peak values from 2013–2015 (**Table 2**).

**Table 2:** Global spatial autocorrelation of PTB distribution in East Gojjam Zone of Ethiopia, 2013–2019.

Year	Moran’s I	Z-score	p-value	Pattern
2013	0.125843	9.71	< 0.001	Clustered
2014	0.132455	10.23	< 0.001	Clustered
2015	0.118259	9.12	< 0.001	Clustered
2016	0.083257	6.42	< 0.001	Clustered
2017	0.076448	6.10	< 0.001	Clustered
2018	0.075917	5.85	< 0.001	Clustered
2019	0.054415	4.24	< 0.001	Clustered

### Purely spatial clusters

**Fig 4:**
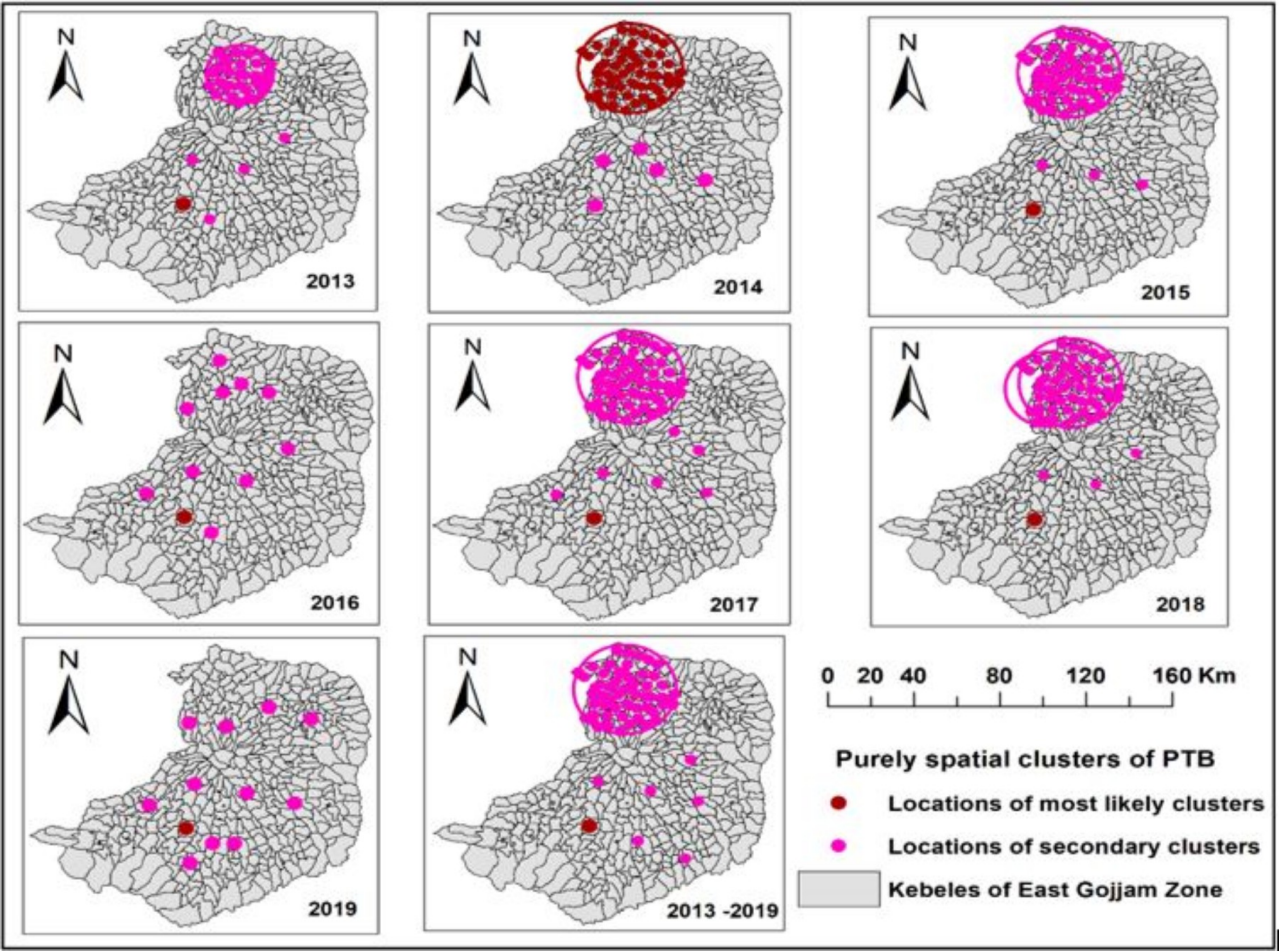
Purely spatial clusters of PTB in the East Gjjam Zone, Northwest Ethiopia, 2013–2019 (Coordinates: 9.900° to 11.193° latitude, and 37.152° to 38.489° longitude).

The purely spatial cluster analysis identified most likely and secondary significant clusters that included a total of 106 cluster locations. Most of the clusters were spotted in the central, northern and northwest parts of the study area. The most likely cluster of PTB cases with 277 observed and 92 expected cases (LLR = 122, p-value < 0.05) were detected at *kebele*s of Debremarkos town administration from 2013–2019. The risk of getting PTB infection among people in *kebeles* of Debremarkos town administration was 3.06 times higher than people residing outside of the town administration. Seven more significant secondary clusters for the high occurrence of PTB cases were detected at *kebeles* of Moseba Shime Abo, Robu Gebeya, kuy, Bichena, Debrework, Lumame, and Dejen town. The first secondary cluster encompassed 56 *Kebeles* (hotspot locations) to which most of the *Kebeles* of Hulet Eju Enese, Goncha Siso Ense, and Bibugn *woredas* are included (**Table 3, Fig 4**).

**Table 3:** Most likely and secondary clusters of purely spatial analysis of PTB cases in the East Gojjam Zone, Northwest Ethiopia, 2013–2019.

Cluster type	Cluster year	Cluster center/radius	Number of cluster locations	Observed cases (n)	Expected cases (n)	LLR	RR	P-value
Most likely cluster	2013–2019	(10.33 N, 37.73E)/0 km	1	277	92	122	3.06	< 0.001
Secondary cluster1	2013–2019	(11.004 N, 37.88 E)/24.6km	56	984	633	89	1.55	< 0.001
Secondary cluster2	2013–2019	(10.55 N, 37.76 E)/0 km	1	69	13	59	5.27	< 0.001
Secondary cluster3	2013–2019	(10.50 N, 37.99 E)/0 km	1	70	15	53	4.72	< 0.001
Secondary cluster4	2013–2019	(10.45 N, 38.20 E)/0 km	1	68	21	34	3.28	< 0.001
Secondary cluster5	2013–2019	(10.66 N, 38.17 E)/0 km	1	65	21	29	3.08	< 0.001
Secondary cluster6	2013–2019	(10.25 N, 37.94 E)/0 km	1	39	13	16	2.95	< 0.001
Secondary cluster7	2013–2019	(10.17 N, 38.15 E)/0 km	1	33	13	12	2.66	< 0.001

Similarly, we evaluated the spatial clusters of PTB cases in each year from 2013–2019 and observed the pattern of most likely and secondary clusters in each year. Most of the cluster locations for the high occurrence of PTB cases were stable over the reporting periods. In addition to one most likely cluster in each year, seven, five, four, ten, six, six, and ten significant secondary spatial clusters were detected during 2013–2019, respectively (**Fig 4, Table 4**).

**Table 4:** Annual purely spatial cluster of PTB cases in East Gojjam Zone, Northwest Ethiopia, 2013–2019.

Cluster type	Cluster year	Cluster center/radius	Number of cluster locations	Observed cases (n)	Expected cases (n)	LLR	RR	P-value
Most likely cluster	2013	(10.33 N, 37.73 E)/0 km	1	68	31	17	2.23	< 0.001
Secondary cluster	2013	(10.50 N, 37.99 E)/0 km	1	23	6	16	4.32	< 0.001
Secondary cluster 1	2013	(10.97 N, 37.97 E)/15.97km	30	196	132	15	1.56	< 0.001
Secondary cluster 2	2013	(10.55 N, 37.76 E)/0 km	1	21	5	14	4.27	< 0.001
Secondary cluster 3	2013	(10.25 N, 37.84 E)/0 km	1	18	4	13	4.40	< 0.001
Secondary cluster 4	2013	(10.97 N, 37.97 E)/0 km	1	40	16	12.7	2.52	< 0.001
Secondary cluster 5	2013	(10.97 N, 37.99 E)/13.15km	20	129	82	12	1.62	< 0.001
Secondary cluster 6	2013	(10.66 N, 38.17 E)/0 km	1	22	8	8	2.78	< 0.001
Most likely cluster	2014	(11.00 N, 37.88 E)/24.46km	56	363	246	29	1.61	< 0.001
Secondary cluster 1	2014	(10.33 N, 37.73 E)/0 km	1	78	37	18	2.19	< 0.001
Secondary cluster 2	2014	(10.55 N, 37.76 E)/0 km	1	22	5	15	4.18	< 0.001
Secondary cluster 3	2014	(10.50 N, 37.99 E)/0 km	1	20	6	11	3.68	< 0.001
Secondary cluster 4	2014	(10.45 N, 38.20 E)/0 km	1	26	9	11	3.04	< 0.001
Secondary cluster 5	2014	(10.61 N, 37.92 E)/0 km	1	16	5	9	3.57	< 0.001
Most likely cluster	2015	(10.33 N, 37.73 E)/0 km	1	80	34	23	2.43	< 0.001
Secondary cluster 1	2015	(10.55 N, 37.76 E)/0 km	1	24	5	18	4.77	< 0.001
Secondary cluster 2	2015	(10.50 N, 37.99 E)/0 km	1	24	6	16	4.26	< 0.001
Secondary cluster 3	2015	(11.004N, 37.88 E)/24.46km	1	315	232	16	1.45	< 0.001
Secondary cluster 4	2015	(10.45 N, 38.20 E)/0 km	1	22	8	8	2.71	< 0.001
Most likely cluster	2016	(10.33 N, 37.73 E)/0 km	1	83	32	30	2.73	< 0.001
Secondary cluster 1	2016	(10.50 N, 37.99 E)/0 km	1	25	5	20	4.91	< 0.001
Secondary cluster 2	2016	(10.55 N, 37.76 E)/0 km	1	23	5	19	5.06	< 0.001
Secondary cluster 3	2016	(10.44 N, 37.57 E)/0 km	1	24	7	13	3.57	< 0.001
Secondary cluster 4	2016	(10.25 N, 37.84 E)/0 km	1	18	4	13	4.53	< 0.001
Secondary cluster 5	2016	(10.97 N, 37.97 E)/0 km	1	18	4	12	4.28	< 0.001
Secondary cluster 6	2016	(11.004 N, 37.88 E)/0 km	1	38	16	11	2.40	< 0.001
Secondary cluster 7	2016	(10.85 N, 37.74E)/0 km	1	18	5	9.7	3.52	< 0.001
Secondary cluster 8	2016	(10.93 N, 37.89 E)/0 km	1	16	4	9.67	3.82	< 0.001
Secondary cluster 9	2016	(10.66 N, 38.17 E)/0 km	1	22	8	9.3	2.98	< 0.001
Secondary cluster10	2016	(10.93 N, 38.09 E)/0 km	1	22	8	9.2	2.96	< 0.001
Most likely cluster	2017	(10.33 N, 37.78 E)/0 km	1	85	29	36	3.04	< 0.001
Secondary cluster 1	2017	(11.004 N, 37.88 E)/24.46km	56	306	207	25	1.61	< 0.001
Secondary cluster 2	2017	(10.50 N, 37.99 E)/0 km	1	21	4	17	5.15	< 0.001
Secondary cluster 3	2017	(10.55 N, 37.76 E)/0 km	1	20	4	15	4.74	< 0.001
Secondary cluster 4	2017	(10.74 N, 38.06 E)/0 km	1	16	4	10	3.94	< 0.001
Secondary cluster 5	2017	(10.66 N, 38.17 E)/0 km	1	22	7	10	3.10	< 0.001
Secondary cluster 6	2017	(10.45 N, 38.20 E)/0 km	1	20	7	9	3.04	< 0.001
Most likely cluster	2018	(10.33 N, 37.73 E)/0 km	1	78	27	33	3.02	< 0.001
Secondary cluster1	2018	(11.004 N, 37.88 E)/24.46km	56	289	193	25	1.63	< 0.001
Secondary cluster 2	2018	(10.97 N, 37.78 E)/20.51 km	35	199	124	21	1.71	< 0.001
Secondary cluster 3	2018	(10.55 N, 37.76 E)/0 km	1	18	4	13	4.62	< 0.001
Secondary cluster 4	2018	(10.50 N, 37.99 E)/0 km	1	18	5	11	3.93	< 0.001
Secondary cluster 5	2018	(10.93 N, 38.09 E)/0 km	1	22	7	11	3.37	< 0.001
Secondary cluster 6	2018	(10.85 N, 38.013E)/0 km	1	15	3	10	4.53	< 0.001
Most likely cluster	2019	(10.33 N, 37.73 E)/0 km	1	114	29	75	4.23	< 0.001
Secondary cluster 1	2019	(10.25 N, 37.84 E)/0 km	1	26	7	28	6.83	< 0.001
Secondary cluster 2	2019	(10.55 N, 37.76 E)/0 km	1	27	5	27	6.27	< 0.001
Secondary cluster 3	2019	(10.85 N, 37.74 E)/0 km	1	27	5	24	5.53	< 0.001
Secondary cluster 4	2019	(10.87 N, 38.27 E)/0 km	1	29	7	19.5	4.22	< 0.001
Secondary cluster 5	2019	(10.45 N, 38.20 E)/0 km	1	28	7	19.4	4.35	< 0.001
Secondary cluster 6	2019	(10.50 N, 37.99 E)/0 km	1	24	5	18.6	4.82	< 0.001
Secondary cluster 7	2019	(10.83 N, 37.90 E)/0 km	1	18	4	15.6	5.40	< 0.001
Secondary cluster8	2019	(10.93 N, 38.09 E)/0 km	1	25	7	15	3.74	< 0.001
Secondary cluster 9	2019	(10.16 N, 37.75 E)/0 km	1	18	5	10.5	3.75	< 0.001
Secondary cluster10	2019	(10.44 N, 37.57 E)/0 km	1	21	7	9.6	3.15	< 0.001

### Spatial-temporal clusters

**Fig 5:**
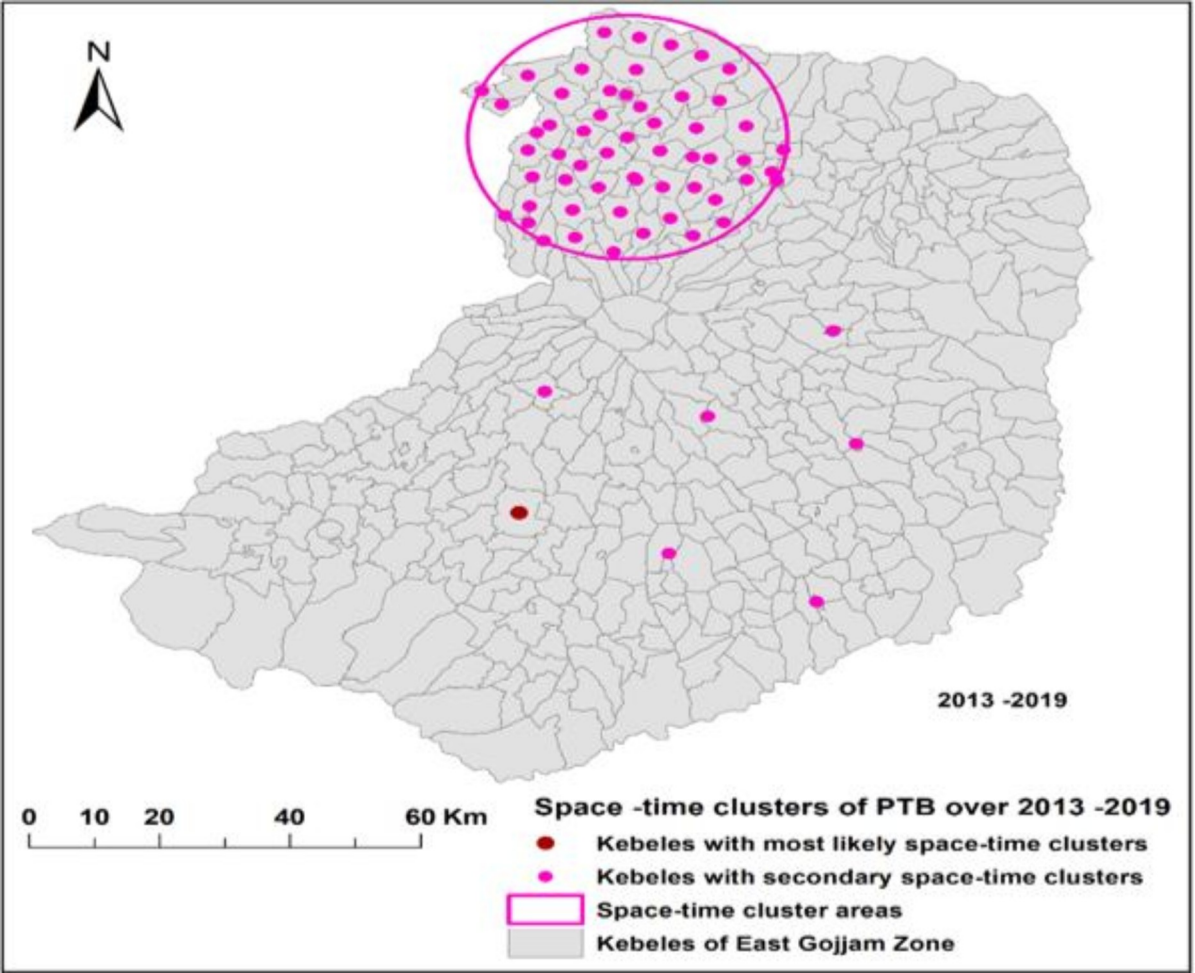
Significant space-time clusters of PTB in East Gojjam Zone of Ethiopia, 2013–2019 (Coordinates: 9.900° to 11.193° latitude, and 37.152° to 38.489° longitude).

A total of eight clusters (one most likely and seven secondary clusters) were detected in 64 cluster locations by space-time cluster analysis. The most likely cluster with 277 observed and 92 expected cases (LLR = 122, p-value < 0.001) was identified from 2017–2019 in *Kebeles* of Debremarkos town Administration. The risk of getting PTB infection in the most likely cluster was 3.06 times higher than people residing outside of the cluster. The first secondary cluster (LLR = 89, p-value < 0.001) detected during the 2013–2015 is comprised of 56 cluster locations in Bibugne, Hulet Eju Enesie and Goncha Siso Enesie woredas that covered 24.46 km cluster radius. The six secondary clusters were from *Kebeles* of Robu Gebeya (2014–2016, RR = 5.27), Kuy (2015–2017, RR = 4.72), Bichena (2013–2015, RR = 3.28), Debrework (2013–2015, RR = 3.08), Lumame (2014–2016, RR = 2.95) and Dejen towns (2013–2015, RR = 2.66). The frequent time frame for the space-time cluster of PTB cases was between 2013–2015 (**Table 5, Fig 5**).

**Table 5:** Space-time clusters of PTB cases in the East Gojjam Zone, Northwest Ethiopia, 2013–2019.

Cluster type	Cluster period	Cluster center/radius	Number of cluster locations	Observed cases	Expected cases	LLR	RR	P-value
Most likely cluster	2017–2019	(10.33 N, 37.73 E)/0 km	1	277	92	122	3.06	< 0.001
Secondary cluster 1	2013–2015	(11.004 N, 37.88E)/24.46 km	56	984	633	89	1.61	< 0.001
Secondary cluster 2	2014–2016	(10.55 N, 37.76 E)/0 km	1	69	13	59	5.27	< 0.001
Secondary cluster 3	2015–2017	(10.50 N, 37.99 E/0 km	1	70	15	53	4.72	< 0.001
Secondary cluster 4	2013–2015	(10.45 N, 38.20 E)/0 km	1	68	21	33.5	3.28	< 0.001
Secondary cluster 5	2013–2015	(10.66 N, 38.17 E)/0 km	1	65	21	29	3.08	< 0.001
Secondary cluster 6	2014–2016	(10.25 N, 37.94 E)/0 km	1	39	13	16	2.95	< 0.001
Secondary cluster 7	2013–2015	(10.17 N, 38.15 E)/0 km	1	33	13	12	2.66	< 0.001

### Purely temporal clusters

From the purely temporal cluster analysis, we identified only one most likely cluster for the high occurrence of PTB from 2013–2015. There were 4959 observed and 4441 expected PTB cases within the cluster and the LLR was 51 (p < 0.001) (F**ig 6**).

**Fig 6:**
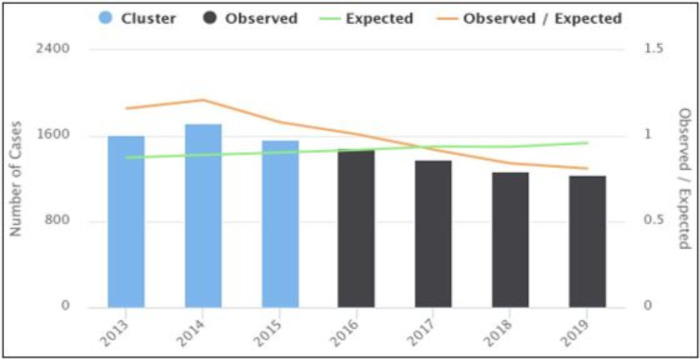
Purely temporal clusters of PTB cases from 2013–2019 in East Gojjam Zone.

### Factors associated with PTB incidence rate at *kebele* levels

Based on negative binomial regression analysis of this study, the incidence rate of PTB for *kebele*s located within 10km distance from the nearest health facility was 0.674 times the incidence rate of PTB for *kebele*s located beyond10km distance holding the other variables constant. The incidence rate of PTB for *kebele*s with health facilities that are ready to offer TB services was 0.663 times the incidence rate of PTB for the counterpart *kebele*s. Similarly, the incidence rate of PTB for rural *kebele*s was 2.221 times the incidence rate of PTB for urban *kebele*s (**Table 6**).

**Table 6:** Factors associated with PTB incidence rates in East Gojjam Zone, Northwest Ethiopia, 2019.

Parameter	Coefficient (B)	Standard error	95% Wald CI		Hypothesis testing			Exp(B)	95% Wald CI of Exp (B)	
			Lower	Upper	Wald chi-square	Df	p-value		Lower	Upper
(Intercept)	3.630	0.2576	3.126	4.135	198.601	1	0.000	37.731	22.773	62.514
Distance from the nearest facility	-0.394	0.0608	-0.514	-0.275	42.085	1	0.000	0.674	0.598	0.759
Readiness of health facility	-0.411	0.0757	-0.560	-0.263	29.502	1	0.000	0.663	0.571	0.769
Population density	0.051	0.1015	-0.148	0.250	0.253	1	0.615	1.052	0.863	1.284
Residence	0.798	0.0781	0.645	0.951	104.443	1	0.000	2.221	1.906	2.589
Scale	1^a^									
Negative binomial	0.153	0.0125	0.130	0.179						

Dependent Variable: TB incidence rate.

Model: (Intercept), distance, Readiness, propensity, residence.

a = Fixed at the displayed value.

## Discussion

Based on the analysis of seven consecutive years of PTB records, the zonal cumulative annual CNR of PTB cases was 58 per 100 000 population ranging from 47 in 2019 to 69 in 2014 per 100 000 population. The CNR of PTB cases varied across districts and *kebele*s. This might be related to low TCD performance due to poor community awareness level, lack of TB diagnostic services, geographic locations, and poor access to transportation facilities [7–9,29,30]. The current CNRs were found to be lower compared to CNRs reported from the Amhara region (107 per 100 000 people) [9], Gurage zone (91.7 per 100 000 people) [10] and Sidama zone (76 per 100 000 people) [8], Ethiopia. This discrepancy is possibly linked to variations of the study period, study area coverage, facility readiness, TB service delays and access to TB diagnostic services [20]. In the study area, healthcare facilities were poorly accessible and only 58.33%(70 of 120) health facilities had TB diagnostic services [21] which are different from the situation reported from southern Ethiopia where 71% and over 80% of health facilities have TB diagnostic services and trained manpower [8,31]. These lead to long patient and health facility delays and low TCD performance [32–34].

The annual CNR of PTB cases showed a decreasing trend from 2015–2019. The decreasing order might be either due to the absence of PTB cases or poor TB detection performance in the study area. In our case, the latter seems true since the PTB CNR of the study area is the least among all zones in the Amhara Region [20]. This is due to the absence of TB diagnostic services in most of the rural health facilities [15,21]. This finding was different from the trends of CNRs in Gurage [10], Sidama [8], Sheka [35], and Arsi [36] zones of Ethiopia, where all showed an increasing trend of CNR across the study periods. This might be linked to variations in study periods, and access and functionality of health facilities to offer TB services. For instance, in the study from the Sidama zone, 71% and over 80% of health facilities had TB diagnostic facilities and trained manpower, respectively. But in our case, very limited health facilities had offered TB diagnostic services and had trained manpower [21].

The CNR of PTB was higher among males (31) than females (26) PTB per 100,000 population. This might be explained by high mobility that leads to having a high chance of acquiring TB infection and relatively better healthcare seeking practices of males. This finding was supported by reports from former studies where CNR was higher among males [9,10,35].

Assessing the spatial-temporal distribution of PTB cases is important to TB prevention and control programs of developing countries including Ethiopia to take targeted anti-TB interventions through proper allocation of scarce resources (budget, manpower, and health facility inputs and supplies) [7,35]. By considering these advantages, we carried out the spatial, temporal and space-time analysis of notified PTB cases at *kebele* levels and the distribution of notified PTB cases was found to be clustered. Based on the SaTScan statistics, most of the most likely and secondary clusters of PTB cases were detected in the central (urban and their surroundings), northern and northwest areas of the zone. The detected PTB clusters were stable over the reporting years. The clustered distribution of PTB cases indicated that there is a need to have targeted anti-TB measures including regular community awareness creation, availing TB diagnostic and treatment services, employing active TCD to prioritized areas, improving TB service quality and avoiding TB service delays [29,30,33].

The distribution of PTB cases is consistent with the former study findings from Southern Ethiopia [8] and the Amhara region [7,9] where most of the PTB clusters were detected in neighboring areas, urban and semi-urban, and neighboring zones/woredas. This might be seen in two ways: firstly, most of the northern parts of the zone are very far from the zonal town and are in the borders of South Wollo and South Gondar zones that have high cross-neighbor population moments than the rest of the districts in the zone. In addition, those areas have large geographic coverage with difficult geographic features (valleys, mountainous and limited transportation infrastructures), high population density with poor income, limited access to health facilities, and low community awareness about TB prevention. Secondly, the central and urban areas including *kebele*s of Debremarkos (zonal town) have relatively better access to TB diagnostic services (public and private). As a result, more people might get TB diagnostic and treatment services. Moreover, these areas have a high population density, transportation facilities, immigration, higher HIV incidence, better access to information sources and more homeless people. These might result in high PTB infection, better healthcare seeking practices and TCD performance [20].

In this study, unlike the former studies [9,10,37], most of the peripheral and southern parts of the study area have relatively lower PTB CNR compared to the northern, central, urban and semi-urban areas. This does not necessarily mean that they have lower PTB incidence cases. Because, we used records of notified PTB patients from people who had better health-seeking practices, access to health facilities and income. There might be more undetected infectious PTB cases in those areas due to low TCD because of low community awareness level, no access to TB diagnostic services, poor transportation facilities, poverty and societal culture and stigma [10,31,38]. In addition, some might be diagnosed and treated for PTB in neighboring health facilities outside of the zone that underestimates the CNR of PTB. Based on our former study in the zone [21], the remote and peripheral areas of the studied zone had poor access to healthcare facilities and those limited health facilities had poor readiness to offer TB diagnostic services. This result was supported by study findings from the Sidama zone [7,8] where the urban and areas around urban had higher CNR of PTB.

The space-time cluster analysis also indicated one more likely and seven secondary clusters of PTB cases in 64 cluster locations with varying time frames during 2013–2019. Four of the eight space-time clusters of PTB were detected in 2013–2015 followed by two clusters in 2014–2017 at different cluster locations. Higher PTB cases were reported during 2013–2015. This implied that anti-TB interventions were not targeted to TB hotspot areas. This might be due to less experience of identifying high TB burden areas, poor TB diagnostic and treatment services, poor monitoring and support of TB control programs, using uniform TCD strategies to all areas, and presence of uncontrolled factors of TB infection in those high TB burden areas [9,10].

Similarly, the purely temporal cluster analysis of this study exhibited only one most likely cluster of PTB cases during 2013–2015. This was similar to the space-time cluster analysis of this study where 50% of space-time clusters were noticed from 2013–2015. This might be explained by the difference in TB control performance, population movements, comorbidities (HIV and DM), facility readiness, and low community awareness level [39–41].

Based on the negative binomial regression analysis of this study, rural residence and distance beyond a 10km radius from the nearest health facility were factors associated with *kebele* level incidence of PTB. This indicated the presence of high PTB transmission and discrepancies in TB control performance between rural and urban areas. Thus, enhanced TB prevention and control activities are required in rural and remote areas [20,42]. This finding was supported by former studies from Ethiopia [4,9,43] and abroad [33,44]. This might be due to poor health service coverage [21,31,33], absence of TB diagnostic services [21,33,45], long delay for TB services (patient and health facility) [32,46–48], limited transportation facilities [21,32,46], and low community awareness level due to poor access to information sources in rural areas [2,30,32,46] compared to urban and surrounding areas.

Poor health facility readiness was also a significant factor for PTB incidence rate at kebele level. This implied that health facilities in the study area had impaired capacity to offer TB services besides their geographic inaccessibility. This was supported by our former study findings from the study area where the overall health service coverage in terms of functional health facilities for TB services was 0.46 per10,000 people and the status of functional sputum smear microscopy service was 2.66 per 100,0000 people. The overall readiness score of health facilities for TB services was 63.5% (95% CI; 25% -101.8%) [21]. The effect of poor health facility readiness on the performance of TB prevention and control programs was also reported by former studies [29,33,46]. It is linked to the absence of core health facility supplies to offer TB services including trained manpower and guidelines, diagnostics, anti TB drugs, and key supplies. This leads to high TB transmission and disease complications, long patient and facility delays by visiting informal healthcare services, and low CNR [20].

The strength of this study relies on including a wider geographic area, analysing large cohorts of PTB cases, using updated population size and geographic shapefiles to each *kebele* and *woreda*, and analysing PTB cases at actual living places (*Kebeles)*. These are important to estimate the burden of PTB and monitor the performance of TB prevention and control programs in the study area.

This study, however, has some limitations. The study findings might not represent the actual PTB status and its spatial-temporal clustering in the zone due to: the zone has low CNR as a result of limited access to and low quality of TB diagnostic services (sputum microscopy), PTB cases with no address information (2.3%) were excluded from the analysis, and our analysis was based upon the notified PTB cases. These might underestimate the magnitude of PTB cases and affect their spatial-temporal clustering in the zone. In addition, there might be some PTB cases who had been screened and attended their treatments outside the zone (in health facilities of neighbouring zones) [32,46,48]. This might also underestimate the magnitude and spatial clustering of PTB cases. Thus, the interpretation and use of the current study findings need to consider these limitations.

## Conclusions

This study identified that PTB cases had a spatial, temporal and space-time clustering pattern at *Kebele* levels during 2013–2019. Based on that, most of the detected PTB clusters were located in the central, northern, and northwest parts of the zone. Moreover, the PTB clustering was more or less stable over the reporting periods. Rural residence, distance from the nearest health facility, and poor TB services readiness were factors of PTB incidence at kebele level. Giving special attention to make health facilities accessible and capable to offer TB services to rural and PTB hotspot areas is crucial to improve CNR and reduce PTB transmission. Using GIS and scan statistics applications, and routine TB data in TB prevention and control activities are vital to monitor and enhance the performance of TB control programs. Also, establishing a cross-neighbor collaboration network might be important in areas with high cross border movements. Conducting further population-based studies is required to investigate the actual PTB status, spatial-temporal clustering, and predictors of PTB infection and clustering.

## Supporting information

S1 File Data for spatial-temporal cluster analysis of PTB cases. (CSV)

S1 Table Characteristics of PTB cases from 2013–2019 in East Gojjam Zone, Ethiopia. (DOCX)

S2 Table Global spatial autocorrelation of PTB distribution in East Gojjam Zone of Ethiopia, 2013–2019. (DOCX)

S3 Table Most likely and secondary clusters of purely spatial analysis of PTB cases in the East Gojjam Zone, Northwest Ethiopia, 2013–2019. (DOCX)

S4 Table Annual purely spatial cluster of PTB cases in East Gojjam Zone, Northwest Ethiopia, 2013–2019. (DOCX)

S5 Table Space-time clusters of PTB cases in the East Gojjam Zone, Northwest Ethiopia, 2013–2019. (DOCX)

S6 Table Factors associated with PTB incidence rates in East Gojjam Zone, Northwest Ethiopia, 2019. (DOCX)
