# Supplementary material for: Spatial-temporal clustering of notified pulmonary tuberculosis and its predictors in East Gojjam Zone, Northwest Ethiopia
Source: PLoS One. 2021 Jan 15;16(1):e0245378. doi: 10.1371/journal.pone.0245378 (PMC7810325; doi:10.1371/journal.pone.0245378)
Supplement: S1 Table — (DOCX) [file pone.0245378.s002.docx]

Tables to PLOS ONE

Table1: Characteristics of PTB cases from 2013 -2019 in East Gojjam Zone, Ethiopia.

| Variable | 2013  N (%) | 2014  N (%) | 2015  N (%) | 2016  N (%) | 2017  N (%) | 2018  N (%) | 2019  N (%) | 2013-2019  N (%) |
| --- | --- | --- | --- | --- | --- | --- | --- | --- |
| Age in years  ≤ 14  15-34  35-44  45+ | 112 (7)  776 (48)  453 (28)  274 (17) | 155 (9)  687 (40)  584 (34)  290 (17) | 110 (7)  704 (45)  423 (27)  327 (21) | 120 (8)  671 (45)  447 (30)  252 (17) | 110 (8)  633 (46)  248 (18)  385 (28) | 127 (10)  633 (50)  304 (24)  203 (16) | 124 (10)  608 (49)  236 (19)  272 (22) | 858(8.3)  4712(46.0)  2695(26.2)  2003(19.5) |
| Sex  Male  Female | 905 (56)  711 (44) | 961 (56)  755 (44) | 860 (55)  704 (45) | 835 (56)  655 (44) | 785 (57)  591 (43) | 696 (55)  569 (45) | 707 (57)  533 (43) | 5751(56.0)  4517(44.0) |
| Residence  Rural  Urban | 873 (54)  743 (46) | 944 (55)  772 (45) | 829 (53)  735 (47) | 805 (54)  685 (46) | 730 (53)  646 (47) | 684 (54)  582 (46) | 682 (55)  558 (45) | 5545(54.0)  4723(46.0) |
| Type of TB  Smear positive PTB  Smear negative PTB | 857 (53)  759 (47) | 893 (52)  823 (48) | 814 (52)  750 (48) | 760 (51)  730 (49) | 716 (52)  660 (48) | 659 (52)  607 (48) | 641(52)  599 (48) | 5340(52.0)  4928(48.0) |
| TB category  New  Retreatment | 1583 (98)  36 (2.2) | 1664 (97)  52 (3) | 1517 (97)  47 (3) | 1460 (98)  30 (2) | 1334 (97)  42 (3.1) | 1236 (98)  28 (2.2) | 1215 (98)  25 (2) | 10008(97.5)  260(2.5) |
| HIV status  Positive  Negative | 178 (11)  1438 (89) | 172 (10)  1544 (90) | 141 (9)  1423 (91) | 134 (9)  1356 (91) | 124 (9)  1252 (92) | 102 (8)  1164 (92) | 87 (7)  1153 (93) | 938(9.0)  9330(91.0) |
| Has TB contact history  Yes  No | 485 (30)  1131 (70) | 549 (32)  1167 (68) | 438 (28)  1126 (72) | 358 (24)  1132 (76) | 248 (18)  1183 (86) | 253 (20)  1013 (80) | 273 (16)  967 (78) | 2604(25.4)  7664(74.6) |
| Treatment outcome  Cured  Completed  Defaulted  Failure  Relapse | 760 (47)  646 (40)  73 (4.5)  75 (6.0)  40 (2.5) | 686 (40)  841 (49)  69 (4.0)  73 (5.0)  34 (2.0) | 657 (42)  750 (48)  63 (4.0)  63 (4.0)  31 (2.0) | 656 (44)  715 (48)  37 (2.5)  56 (4.0)  22 (1.5) | 601 (42)  716 (50)  43 (3.0)  48 (4.0)  14 (1.0) | 532 (42)  646 (51)  38 (3.0)  31 (2.5)  19 (1.5) | 546 (44)  620 (50)  25 (2.0)  30 (3.0)  12 (1.0) | 4438(43.2)  4934(48.0)  348(3.4)  376(3.7)  172(1.7) |
| Total | 1594 | 1703 | 1564 | 1486 | 1422 | 1266 | 1233 | 10,268 |
