# Supplementary material for: Spatial-temporal clustering of notified pulmonary tuberculosis and its predictors in East Gojjam Zone, Northwest Ethiopia
Source: PLoS One. 2021 Jan 15;16(1):e0245378. doi: 10.1371/journal.pone.0245378 (PMC7810325; doi:10.1371/journal.pone.0245378)
Supplement: S2 Table — (DOCX) [file pone.0245378.s003.docx]

Table2: Global spatial autocorrelation of PTB distribution in East Gojjam Zone of Ethiopia, 2013-2019.

| Year | Moran’s I | Z-score | p-value | Pattern | |
| --- | --- | --- | --- | --- | --- |
| 2013 | 0.125843 | 9.71 | < 0.001 | Clustered | |
| 2014 | 0.132455 | 10.23 | < 0.001 | Clustered | |
| 2015 | 0.118259 | 9.12 | < 0.001 | Clustered | |
| 2016 | 0.083257 | 6.42 | < 0.001 | Clustered | |
|  |  |  |  |  | |
| 2017 | 0.076448 | 6.10 | < 0.001 | Clustered | |
| 2018 | 0.075917 | 5.85 | < 0.001 | Clustered | |
| 2019 | 0.054415 | 4.24 | < 0.001 | | Clustered |
