# Supplementary material for: Spatial-temporal clustering of notified pulmonary tuberculosis and its predictors in East Gojjam Zone, Northwest Ethiopia
Source: PLoS One. 2021 Jan 15;16(1):e0245378. doi: 10.1371/journal.pone.0245378 (PMC7810325; doi:10.1371/journal.pone.0245378)
Supplement: S3 Table — (DOCX) [file pone.0245378.s004.docx]

Table3. Most likely and secondary clusters of purely spatial analysis of PTB cases in the East Gojjam Zone, Northwest Ethiopia, 2013-2019.

| Cluster type | Cluster year | Cluster center/radius | Number of cluster locations | Observed cases (n) | Expected cases (n) | LLR | RR | P-value |
| --- | --- | --- | --- | --- | --- | --- | --- | --- |
| Most likely cluster | 2013-2019 | (10.33 N, 37.73E) / 0 km | 1 | 277 | 92 | 122 | 3.06 | < 0.001 |
| Secondary cluster1 | 2013-2019 | (11.004 N, 37.88 E)/24.6km | 56 | 984 | 633 | 89 | 1.55 | < 0.001 |
| Secondary cluster2 | 2013-2019 | (10.55 N, 37.76 E) / 0 km | 1 | 69 | 13 | 59 | 5.27 | < 0.001 |
| Secondary cluster3 | 2013-2019 | (10.50 N, 37.99 E) / 0 km | 1 | 70 | 15 | 53 | 4.72 | < 0.001 |
| Secondary cluster4 | 2013-2019 | (10.45 N, 38.20 E) / 0 km | 1 | 68 | 21 | 34 | 3.28 | < 0.001 |
| Secondary cluster5 | 2013-2019 | (10.66 N, 38.17 E) / 0 km | 1 | 65 | 21 | 29 | 3.08 | < 0.001 |
| Secondary cluster6 | 2013-2019 | (10.25 N, 37.94 E) / 0 km | 1 | 39 | 13 | 16 | 2.95 | < 0.001 |
| Secondary cluster7 | 2013-2019 | (10.17 N, 38.15 E) / 0 km | 1 | 33 | 13 | 12 | 2.66 | < 0.001 |
