# Supplementary material for: Spatial-temporal clustering of notified pulmonary tuberculosis and its predictors in East Gojjam Zone, Northwest Ethiopia
Source: PLoS One. 2021 Jan 15;16(1):e0245378. doi: 10.1371/journal.pone.0245378 (PMC7810325; doi:10.1371/journal.pone.0245378)
Supplement: S4 Table — (DOCX) [file pone.0245378.s005.docx]

Table4. Annual purely spatial cluster of PTB cases in East Gojjam Zone, Northwest Ethiopia, 2013-2019.

| Cluster type | Cluster year | Cluster center/radius | Number of cluster locations | Observed cases (n) | Expected cases (n) | LLR | RR | P-value |
| --- | --- | --- | --- | --- | --- | --- | --- | --- |
| Most likely cluster | 2013 | (10.33 N, 37.73 E) / 0 km | 1 | 68 | 31 | 17 | 2.23 | < 0.001 |
| Secondary cluster | 2013 | (10.50 N, 37.99 E) / 0 km | 1 | 23 | 6 | 16 | 4.32 | < 0.001 |
| Secondary cluster 1 | 2013 | (10.97 N, 37.97 E)/15.97km | 30 | 196 | 132 | 15 | 1.56 | < 0.001 |
| Secondary cluster 2 | 2013 | (10.55 N, 37.76 E) / 0 km | 1 | 21 | 5 | 14 | 4.27 | < 0.001 |
| Secondary cluster 3 | 2013 | (10.25 N, 37.84 E) / 0 km | 1 | 18 | 4 | 13 | 4.40 | < 0.001 |
| Secondary cluster 4 | 2013 | (10.97 N, 37.97 E) / 0 km | 1 | 40 | 16 | 12.7 | 2.52 | < 0.001 |
| Secondary cluster 5 | 2013 | (10.97 N, 37.99 E)/13.15km | 20 | 129 | 82 | 12 | 1.62 | < 0.001 |
| Secondary cluster 6 | 2013 | (10.66 N, 38.17 E) / 0 km | 1 | 22 | 8 | 8 | 2.78 | < 0.001 |
| Most likely cluster | 2014 | (11.00 N, 37.88 E)/24.46km | 56 | 363 | 246 | 29 | 1.61 | < 0.001 |
| Secondary cluster 1 | 2014 | (10.33 N, 37.73 E) / 0 km | 1 | 78 | 37 | 18 | 2.19 | < 0.001 |
| Secondary cluster 2 | 2014 | (10.55 N, 37.76 E) / 0 km | 1 | 22 | 5 | 15 | 4.18 | < 0.001 |
| Secondary cluster 3 | 2014 | (10.50 N, 37.99 E) / 0 km | 1 | 20 | 6 | 11 | 3.68 | < 0.001 |
| Secondary cluster 4 | 2014 | (10.45 N, 38.20 E) / 0 km | 1 | 26 | 9 | 11 | 3.04 | < 0.001 |
| Secondary cluster 5 | 2014 | (10.61 N, 37.92 E) / 0 km | 1 | 16 | 5 | 9 | 3.57 | < 0.001 |
| Most likely cluster | 2015 | (10.33 N, 37.73 E) / 0 km | 1 | 80 | 34 | 23 | 2.43 | < 0.001 |
| Secondary cluster 1 | 2015 | (10.55 N, 37.76 E) / 0 km | 1 | 24 | 5 | 18 | 4.77 | < 0.001 |
| Secondary cluster 2 | 2015 | (10.50 N, 37.99 E) / 0 km | 1 | 24 | 6 | 16 | 4.26 | < 0.001 |
| Secondary cluster 3 | 2015 | (11.004N, 37.88 E)/24.46km | 1 | 315 | 232 | 16 | 1.45 | < 0.001 |
| Secondary cluster 4 | 2015 | (10.45 N, 38.20 E) / 0 km | 1 | 22 | 8 | 8 | 2.71 | < 0.001 |
| Most likely cluster | 2016 | (10.33 N, 37.73 E) / 0 km | 1 | 83 | 32 | 30 | 2.73 | < 0.001 |
| Secondary cluster 1 | 2016 | (10.50 N, 37.99 E) / 0 km | 1 | 25 | 5 | 20 | 4.91 | < 0.001 |
| Secondary cluster 2 | 2016 | (10.55 N, 37.76 E) / 0 km | 1 | 23 | 5 | 19 | 5.06 | < 0.001 |
| Secondary cluster 3 | 2016 | (10.44 N, 37.57 E) / 0 km | 1 | 24 | 7 | 13 | 3.57 | < 0.001 |
| Secondary cluster 4 | 2016 | (10.25 N, 37.84 E) / 0 km | 1 | 18 | 4 | 13 | 4.53 | < 0.001 |
| Secondary cluster 5 | 2016 | (10.97 N, 37.97 E) / 0 km | 1 | 18 | 4 | 12 | 4.28 | < 0.001 |
| Secondary cluster 6 | 2016 | (11.004 N, 37.88 E)/0 km | 1 | 38 | 16 | 11 | 2.40 | < 0.001 |
| Secondary cluster 7 | 2016 | (10.85 N, 37.74E) / 0 km | 1 | 18 | 5 | 9.7 | 3.52 | < 0.001 |
| Secondary cluster 8 | 2016 | (10.93 N, 37.89 E) / 0 km | 1 | 16 | 4 | 9.67 | 3.82 | < 0.001 |
| Secondary cluster 9 | 2016 | (10.66 N, 38.17 E) / 0 km | 1 | 22 | 8 | 9.3 | 2.98 | < 0.001 |
| Secondary cluster10 | 2016 | (10.93 N, 38.09 E) / 0 km | 1 | 22 | 8 | 9.2 | 2.96 | < 0.001 |
| Most likely cluster | 2017 | (10.33 N, 37.78 E) / 0 km | 1 | 85 | 29 | 36 | 3.04 | < 0.001 |
| Secondary cluster 1 | 2017 | (11.004 N, 37.88 E)/24.46km | 56 | 306 | 207 | 25 | 1.61 | < 0.001 |
| Secondary cluster 2 | 2017 | (10.50 N, 37.99 E) / 0 km | 1 | 21 | 4 | 17 | 5.15 | < 0.001 |
| Secondary cluster 3 | 2017 | (10.55 N, 37.76 E) / 0 km | 1 | 20 | 4 | 15 | 4.74 | < 0.001 |
| Secondary cluster 4 | 2017 | (10.74 N, 38.06 E) / 0 km | 1 | 16 | 4 | 10 | 3.94 | < 0.001 |
| Secondary cluster 5 | 2017 | (10.66 N, 38.17 E) / 0 km | 1 | 22 | 7 | 10 | 3.10 | < 0.001 |
| Secondary cluster 6 | 2017 | (10.45 N, 38.20 E) / 0 km | 1 | 20 | 7 | 9 | 3.04 | < 0.001 |
| Most likely cluster | 2018 | (10.33 N, 37.73 E) / 0 km | 1 | 78 | 27 | 33 | 3.02 | < 0.001 |
| Secondary cluster1 | 2018 | (11.004 N, 37.88 E)/24.46km | 56 | 289 | 193 | 25 | 1.63 | < 0.001 |
| Secondary cluster 2 | 2018 | (10.97 N, 37.78 E)/20.51 km | 35 | 199 | 124 | 21 | 1.71 | < 0.001 |
| Secondary cluster 3 | 2018 | (10.55 N, 37.76 E) / 0 km | 1 | 18 | 4 | 13 | 4.62 | < 0.001 |
| Secondary cluster 4 | 2018 | (10.50 N, 37.99 E) / 0 km | 1 | 18 | 5 | 11 | 3.93 | < 0.001 |
| Secondary cluster 5 | 2018 | (10.93 N, 38.09 E) / 0 km | 1 | 22 | 7 | 11 | 3.37 | < 0.001 |
| Secondary cluster 6 | 2018 | (10.85 N, 38.013E) / 0 km | 1 | 15 | 3 | 10 | 4.53 | < 0.001 |
| Most likely cluster | 2019 | (10.33 N, 37.73 E) / 0 km | 1 | 114 | 29 | 75 | 4.23 | < 0.001 |
| Secondary cluster 1 | 2019 | (10.25 N, 37.84 E) / 0 km | 1 | 26 | 7 | 28 | 6.83 | < 0.001 |
| Secondary cluster 2 | 2019 | (10.55 N, 37.76 E) / 0 km | 1 | 27 | 5 | 27 | 6.27 | < 0.001 |
| Secondary cluster 3 | 2019 | (10.85 N, 37.74 E) / 0 km | 1 | 27 | 5 | 24 | 5.53 | < 0.001 |
| Secondary cluster 4 | 2019 | (10.87 N, 38.27 E) / 0 km | 1 | 29 | 7 | 19.5 | 4.22 | < 0.001 |
| Secondary cluster 5 | 2019 | (10.45 N, 38.20 E) / 0 km | 1 | 28 | 7 | 19.4 | 4.35 | < 0.001 |
| Secondary cluster 6 | 2019 | (10.50 N, 37.99 E) / 0 km | 1 | 24 | 5 | 18.6 | 4.82 | < 0.001 |
| Secondary cluster 7 | 2019 | (10.83 N, 37.90 E) / 0 km | 1 | 18 | 4 | 15.6 | 5.40 | < 0.001 |
| Secondary cluster8 | 2019 | (10.93 N, 38.09 E) / 0 km | 1 | 25 | 7 | 15 | 3.74 | < 0.001 |
| Secondary cluster 9 | 2019 | (10.16 N, 37.75 E) / 0 km | 1 | 18 | 5 | 10.5 | 3.75 | < 0.001 |
| Secondary cluster10 | 2019 | (10.44 N, 37.57 E) / 0 km | 1 | 21 | 7 | 9.6 | 3.15 | < 0.001 |
