# Supplementary material for: Spatial-temporal clustering of notified pulmonary tuberculosis and its predictors in East Gojjam Zone, Northwest Ethiopia
Source: PLoS One. 2021 Jan 15;16(1):e0245378. doi: 10.1371/journal.pone.0245378 (PMC7810325; doi:10.1371/journal.pone.0245378)
Supplement: S5 Table — (DOCX) [file pone.0245378.s006.docx]

Table5. Space-time clusters of PTB cases in the East Gojjam Zone, Northwest Ethiopia, 2013–2019.

| Cluster type | Cluster period | Cluster center/radius | Number of cluster locations | Observed cases | Expected cases | LLR | RR | P-value |
| --- | --- | --- | --- | --- | --- | --- | --- | --- |
| Most likely cluster | 2017-2019 | (10.33 N, 37.73 E)/0 km | 1 | 277 | 92 | 122 | 3.06 | < 0.001 |
| Secondary cluster 1 | 2013-2015 | (11.004 N, 37.88E)/24.46 km | 56 | 984 | 633 | 89 | 1.61 | < 0.001 |
| Secondary cluster 2 | 2014-2016 | (10.55 N, 37.76 E)/ 0 km | 1 | 69 | 13 | 59 | 5.27 | < 0.001 |
| Secondary cluster 3 | 2015-2017 | (10.50 N, 37.99 E/ 0 km | 1 | 70 | 15 | 53 | 4.72 | < 0.001 |
| Secondary cluster 4 | 2013-2015 | (10.45 N, 38.20 E)/ 0 km | 1 | 68 | 21 | 33.5 | 3.28 | < 0.001 |
| Secondary cluster 5 | 2013-2015 | (10.66 N, 38.17 E)/ 0 km | 1 | 65 | 21 | 29 | 3.08 | < 0.001 |
| Secondary cluster 6 | 2014-2016 | (10.25 N, 37.94 E)/ 0 km | 1 | 39 | 13 | 16 | 2.95 | < 0.001 |
| Secondary cluster 7 | 2013-2015 | (10.17 N, 38.15 E)/ 0 km | 1 | 33 | 13 | 12 | 2.66 | < 0.001 |
