# Supplementary material for: Spatial-temporal clustering of notified pulmonary tuberculosis and its predictors in East Gojjam Zone, Northwest Ethiopia
Source: PLoS One. 2021 Jan 15;16(1):e0245378. doi: 10.1371/journal.pone.0245378 (PMC7810325; doi:10.1371/journal.pone.0245378)
Supplement: S6 Table — (DOCX) [file pone.0245378.s007.docx]

Table6. Factors associated with PTB incidence rates in East Gojjam Zone, Northwest Ethiopia, 2019.

| Parameter | Coefficient (B) | Standard error | 95% Wald CI | | Hypothesis testing | | | Exp(B) | 95% Wald CI of Exp (B) | |
| --- | --- | --- | --- | --- | --- | --- | --- | --- | --- | --- |
|  |  |  | Lower | Upper | Wald chi-square | Df | p-value |  | Lower | Upper |
| (Intercept) | 3.630 | 0.2576 | 3.126 | 4.135 | 198.601 | 1 | 0.000 | 37.731 | 22.773 | 62.514 |
| Distance from the nearest facility | -0.394 | 0.0608 | -0.514 | -0.275 | 42.085 | 1 | 0.000 | 0.674 | 0.598 | 0.759 |
| Readiness of health facility | -0.411 | 0.0757 | -0.560 | -0.263 | 29.502 | 1 | 0.000 | 0.663 | 0.571 | 0.769 |
| Population density | 0.051 | 0.1015 | -0.148 | 0.250 | 0.253 | 1 | 0.615 | 1.052 | 0.863 | 1.284 |
| Residence | 0.798 | 0.0781 | 0.645 | 0.951 | 104.443 | 1 | 0.000 | 2.221 | 1.906 | 2.589 |
| Scale | 1^a^ |  |  |  |  |  |  |  |  |  |
| Negative binomial | 0.153 | 0.0125 | 0.130 | 0.179 |  |  |  |  |  |  |
